# Supplementary material for: The Acquisition of Colistin Resistance Is Associated to the Amplification of a Large Chromosomal Region in Klebsiella pneumoniae kp52145
Source: Int J Mol Sci. 2021 Jan 11;22(2):649. doi: 10.3390/ijms22020649 (PMC7826664; doi:10.3390/ijms22020649)
Supplement: Supplementary file 1 [file ijms-22-00649-s001.pdf]

Table S1 Data obtained from the PHAge Search Tool Enhanced Release of the present prophage sequence regions in *K. pneumoniae* kp52145.

Prophage 1

CD position	BLAST-HIT
1506010..1506048	attL
complement(1506070..1507233)	PHAGE_Escher_HK639_NC_016158: integrase; BN49_RS08730; phage(gi356870629)
complement(1507110..1507445)	PHAGE_Cronob_ENT47670_NC_019927: excisionase; BN49_RS28755; phage(gi431810536)
complement(1507447..1507665)	PHAGE_Stx2_vB_EcoP_24B_NC_027984: putative C4 zinc finger protein; BN49_RS08735; phage(gi937456240)
complement(1507662..1508039)	DUF2591 domain-containing protein; BN49_RS08740
complement(1508036..1508758)	PHAGE_Salmon_SJ46_NC_031129: integrase/recombinase; BN49_RS08745; phage(gi100097)
complement(1508755..1508976)	PHAGE_Entero_BP_4795_NC_004813: hypothetical protein; BN49_RS08750; phage(gi157165994)
complement(1508973..1509500)	PHAGE_Entero_933W_NC_000924: putative DNA N-6-adenine-methyltransferase; BN49_RS28050; phage(gi9632500)
complement(1509497..1509655)	PHAGE_Entero_VT2_Sakai_NC_000902: hypothetical protein; BN49_RS28760; phage(gi9633408)
complement(1509652..1510332)	PHAGE_Salmon_SEN34_NC_028699: exonuclease; BN49_RS08765; phage(gi966201454)
complement(1510329..1511174)	PHAGE_Ralsto_RSK1_NC_022915: putative phage recombination protein; BN49_RS08770; phage(gi560186064)
complement(1511190..1511474)	PHAGE_Escher_HK639_NC_016158: hypothetical protein; BN49_RS08775; phage(gi356870640)
complement(1511509..1511712)	PHAGE_Entero_phi80_NC_021190: anti-toxin for Gam; BN49_RS08780; phage(gi824479616)
complement(1511796..1512311)	PHAGE_Salmon_IME207_NC_031924: hypothetical protein; BN49_RS08785; phage(gi100002)
complement(1512308..1512523)	PHAGE_Salmon_SEN34_NC_028699: membrane carboxypeptidase; BN49_RS28765; phage(gi966201457)
1512961..1513164	PHAGE_Salmon_118970_sal4_NC_030919: hypothetical protein; BN49_RS08795; phage(gi100018)
complement(1513205..1514125)	hypothetical protein; BN49_RS08800
complement(1514204..1514902)	PHAGE_Salmon_ST160_NC_014900: C2; BN49_RS08805; phage(gi318065925)
1515014..1515241	PHAGE_Salmon_ST160_NC_014900: Cro; BN49_RS08810; phage(gi318065926)
1515282..1515566	PHAGE_Entero_mEp213_NC_019720: CII protein; BN49_RS08815; phage(gi428782643)
1515601..1516470	PHAGE_Entero_mEpX1_NC_019709: DNA replication protein O; BN49_RS08820; phage(gi428781918)
1516473..1517324	PHAGE_Entero_mEpX1_NC_019709: putative replication protein DnaC; BN49_RS08825; phage(gi428781919)
1517321..1517614	PHAGE_Entero_933W_NC_000924: Ren protein; BN49_RS08830; phage(gi9632496)
1517611..1518081	PHAGE_Vibrio_pYD38_A_NC_021534: hypothetical protein; BN49_RS08835; phage(gi514051016)
1518078..1518440	PHAGE_Entero_ES18_NC_006949: gp42; BN49_RS08840; phage(gi62362255)
1518437..1519165	PHAGE_Vibrio_12A4_NC_021068: DNA methylase; BN49_RS08845; phage(gi481019118)
1519447..1519734	PHAGE_Entero_mEp390_NC_019721: hypothetical protein; BN49_RS29935; phage(gi428782697)
1519734..1519922	PHAGE_Salmon_118970_sal4_NC_030919: kilA anti-repressor protein; BN49_RS08855; phage(gi100007)
1520200..1520382	PHAGE_Salmon_SETP13_NC_022752: EaA protein; BN49_RS28770; phage(gi557307718)
1520826..1521131	PHAGE_Entero_mEp390_NC_019721: hypothetical protein; BN49_RS08865; phage(gi428782694)
1521383..1521850	PHAGE_Stx2_c_1717_NC_011357: NinB protein; BN49_RS08870; phage(gi209447158)
1521831..1522001	PHAGE_Escher_HK639_NC_016158: NinE; BN49_RS28775; phage(gi356870663)
1521994..1522629	PHAGE_Entero_phi80_NC_021190: DNA junction-specific endonuclease Rap; BN49_RS08880; phage(gi824479632)
1522756..1523286	PHAGE_Entero_Tyrion_NC_031077: hypothetical protein; BN49_RS08885; phage(gi100047)
1523283..1523972	PHAGE_Phage_Gifsy_1_NC_010392: bacteriophage antiterminator protein Q; BN49_RS08890; phage(gi169257244)
1524075..1524149	tRNA
1524155..1524231	tRNA

1524788..1525102	PHAGE_Salmon_SPN3UB_NC_019545: hypothetical protein; BN49_RS08905; phage(gi423262443)
1525105..1525608	PHAGE_Salmon_118970_sal4_NC_030919: major capsid protein; BN49_RS08910; phage(gi100039)
1525709..1526086	PHAGE_Entero_SfV_NC_003444: putative Rz lytic protein; BN49_RS08915; phage(gi19549038)
1526067..1526267	PHAGE_Shigel_SfIV_NC_022749: o-spanin (Rz1); BN49_RS28785; phage(gi557307579)
1526710..1527345	PHAGE_Pectob_ZF40_NC_019522: putative transposase; BN49_RS08920; phage(gi422936679)
1527378..1527857	PHAGE_Pseudo_JBD44_NC_030929: hypothetical protein; BN49_RS08925; phage(gi100017)
1527844..1529316	PHAGE_Vibrio_pYD38_B_NC_021561: hypothetical protein; BN49_RS08930; phage(gi514231577)
1529328..1530776	PHAGE_Salmon_64795_sal3_NC_031918: hypothetical protein; BN49_RS08935; phage(gi100003)
1530712..1531710	PHAGE_Salmon_IME207_NC_031924: endonuclease; BN49_RS08940; phage(gi100011)
1531940..1533295	PHAGE_Cronob_ENT47670_NC_019927: hypothetical protein; BN49_RS08945; phage(gi431810499)
1533295..1533756	PHAGE_Salmon_IME207_NC_031924: hypothetical protein; BN49_RS08950; phage(gi100042)
1533753..1534808	PHAGE_Salmon_IME207_NC_031924: hypothetical protein; BN49_RS08955; phage(gi100043)
1534841..1535197	hypothetical protein; BN49_RS08960
1535200..1535580	PHAGE_Cronob_ENT47670_NC_019927: hypothetical protein; BN49_RS08965; phage(gi431810531)
1535580..1535753	PHAGE_Salmon_vB_SosS_Oslo_NC_018279: hypothetical protein; BN49_RS08970; phage(gi399528768)
1535753..1536115	PHAGE_Salmon_64795_sal3_NC_031918: hypothetical protein; BN49_RS08975; phage(gi100041)
1536118..1536486	PHAGE_Salmon_64795_sal3_NC_031918: hypothetical protein; BN49_RS08980; phage(gi100042)
1536483..1536866	PHAGE_Salmon_64795_sal3_NC_031918: hypothetical protein; BN49_RS08985; phage(gi100044)
1536925..1537689	PHAGE_Salmon_64795_sal3_NC_031918: hypothetical protein; BN49_RS08990; phage(gi100045)
1537758..1538447	PHAGE_Salmon_64795_sal3_NC_031918: tail tape measure protein; BN49_RS08995; phage(gi100049)
1538758..1539330	PHAGE_Shigel_Stx_NC_029120: hypothetical protein; BN49_RS09000; phage(gi985761316)
1539327..1539779	hypothetical protein; BN49_RS28790
complement(1539822..1540244)	PHAGE_Pseudo_YMC11/07/P54_PAE_BP_NC_030909: tail tape measure protein; BN49_RS09005; phage(gi100049)
complement(1540292..1541155)	PHAGE_Entero_phiP27_NC_003356: putative lambda repressor; BN49_RS09010; phage(gi18249875)
1541504..1542388	PHAGE_Salmon_vB_SosS_Oslo_NC_018279: putative antirepressor protein Ant; BN49_RS09015; phage(gi399528777)
1542458..1543210	PHAGE_Salmon_SJ46_NC_031129: hypothetical protein; BN49_RS09020; phage(gi100021)
1543398..1543730	hypothetical protein; BN49_RS09025
1543727..1544200	hypothetical protein; BN49_RS28795
1544245..1546662	PHAGE_Cronob_ENT47670_NC_019927: putative tail protein; BN49_RS09030; phage(gi431810494)
complement(1546811..1548084)	PHAGE_Pseudo_MD8_NC_031091: hypothetical protein; BN49_RS09035; phage(gi100010)
1548285..1548464	hypothetical protein; BN49_RS09045
complement(1548441..1548707)	hypothetical protein; BN49_RS28800
1548821..1549297	PHAGE_Cronob_ENT47670_NC_019927: hypothetical protein; BN49_RS09050; phage(gi431810519)
1549297..1549767	PHAGE_Vibrio_pYD38_A_NC_021534: hypothetical protein; BN49_RS09055; phage(gi514051035)
1549764..1550159	PHAGE_Salmon_II_E1_NC_010495: possible tail assembly protein; BN49_RS09060; phage(gi170676313)
1550146..1552617	PHAGE_Cronob_ENT47670_NC_019927: putative tail protein; BN49_RS09065; phage(gi431810495)
1552704..1554887	PHAGE_Burkho_KS10_NC_011216: hypothetical protein; BN49_RS09070; phage(gi198449310)
1554900..1555634	PHAGE_Burkho_KS10_NC_011216: hypothetical protein; BN49_RS09075; phage(gi198449311)
1555653..1555859	PHAGE_Entero_mEp390_NC_019721: hypothetical protein; BN49_RS09080; phage(gi428782690)
1556027..1556065	attR

Prophage 2

CD position	BLAST-HIT
2011787..2011805	attL
complement(2011880..2012932)	PHAGE_Salmon_Fels_2_NC_010463: integrase; BN49_RS11210; phage(gi169936064)
complement(2013019..2014008)	hypothetical protein; BN49_RS11215
complement(2014019..2014957)	PHAGE_Salmon_Fels_2_NC_010463: P2 CI-like protein; BN49_RS28910; phage(gi169936063)
2015046..2015267	PHAGE_Haemop_HP2_NC_003315: orf2(S)cox; BN49_RS28915; phage(gi17981819)
2015300..2015809	PHAGE_Salmon_Fels_2_NC_010463: bacteriophage regulatory protein CII; BN49_RS11225; phage(gi169936061)
2015817..2016017	PHAGE_Salmon_Fels_2_NC_010463: hypothetical protein; BN49_RS11230; phage(gi169936060)
2015981..2016322	PHAGE_Salmon_Fels_2_NC_010463: hypothetical protein; BN49_RS11235; phage(gi169936058)
2016390..2016623	PHAGE_Salmon_Fels_2_NC_010463: hypothetical protein; BN49_RS11240; phage(gi169936057)
2016623..2016850	PHAGE_Salmon_Fels_2_NC_010463: DksA-like zinc finger domain containing protein; BN49_RS11245; phage(gi169936056)
2016847..2017704	PHAGE_Salmon_Fels_2_NC_010463: DNA adenine methylase-like protein; BN49_RS11250; phage(gi169936055)
2017701..2020115	PHAGE_Salmon_Fels_2_NC_010463: P2 gpA-like protein; BN49_RS11255; phage(gi169936054)
2020269..2020457	PHAGE_Salmon_Fels_2_NC_010463: hypothetical protein; BN49_RS11260; phage(gi169936053)
2020468..2020701	PHAGE_Salmon_Fels_2_NC_010463: TumB protein; BN49_RS11265; phage(gi169936052)
2020988..2021206	PHAGE_Salmon_SEN5_NC_028701: hypothetical protein; BN49_RS11270; phage(gi966201554)
2021206..2022048	PHAGE_Salmon_SEN5_NC_028701: hypothetical protein; BN49_RS11275; phage(gi966201555)
2022058..2022267	hypothetical protein; BN49_RS11280
2022264..2023697	SEFIR domain-containing protein; BN49_RS11285
complement(2023732..2024772)	PHAGE_Salmon_Fels_2_NC_010463: portal vertex protein; BN49_RS11290; phage(gi169936049)
complement(2024769..2025494)	PHAGE_Entero_186_NC_001317: W protein; BN49_RS11295; phage(gi9634057)
complement(2025494..2027260)	PHAGE_Salmon_Fels_2_NC_010463: terminase ATPase subunit; BN49_RS11300; phage(gi169936048)
2027403..2028236	PHAGE_Salmon_Fels_2_NC_010463: capsid-scaffolding protein; BN49_RS11305; phage(gi169936047)
2028253..2029311	PHAGE_Salmon_Fels_2_NC_010463: capsid protein; BN49_RS11310; phage(gi169936046)
2029315..2029965	PHAGE_Salmon_Fels_2_NC_010463: terminase endonuclease subunit; BN49_RS11315; phage(gi169936045)
2030061..2030525	PHAGE_Salmon_Fels_2_NC_010463: P2 gpL-like protein; BN49_RS11320; phage(gi169936044)
2030525..2030728	PHAGE_Salmon_Fels_2_NC_010463: P2 gpX-like tail protein; BN49_RS11325; phage(gi169936043)
2030732..2030947	PHAGE_Salmon_Fels_2_NC_010463: lysis protein; BN49_RS11330; phage(gi169936042)
2030928..2031440	PHAGE_Salmon_Fels_2_NC_010463: endolysin; BN49_RS11335; phage(gi169936041)
2031442..2031819	hypothetical protein; BN49_RS11340
2031816..2032244	PHAGE_Salmon_Fels_2_NC_010463: P2 LysB-like protein; BN49_RS11345; phage(gi169936038)
2032319..2032771	PHAGE_Salmon_Fels_2_NC_010463: P2 gpR-like tail completion protein; BN49_RS11355; phage(gi169936036)
2032764..2033210	PHAGE_Salmon_Fels_2_NC_010463: P2 gpS-like tail completion protein; BN49_RS11360; phage(gi169936035)
2033279..2033857	PHAGE_Salmon_Fels_2_NC_010463: P2 gpV-like protein; BN49_RS11365; phage(gi169936034)
2033854..2034213	PHAGE_Salmon_Fels_2_NC_010463: baseplate wedge subunit; BN49_RS11370; phage(gi169936033)
2034200..2035108	PHAGE_Salmon_Fels_2_NC_010463: baseplate assembly protein; BN49_RS11375; phage(gi169936032)
2035101..2035706	PHAGE_Salmon_Fels_2_NC_010463: P2 gpI-like baseplate assembly protein; BN49_RS11380; phage(gi169936031)
2035703..2037424	PHAGE_Salmon_Fels_2_NC_010463: P2 gpH-like protein; BN49_RS11385; phage(gi169936030)
2037424..2037606	hypothetical protein; BN49_RS11390
complement(2037587..2037739)	PHAGE_Shigel_SfIV_NC_022749: tail fiber assembly protein; BN49_RS28920; phage(gi557307547)

complement(2037760..2038207)	PHAGE_Shigel_SfIV_NC_022749: IS1 transposase B; BN49_RS11395; phage(gi557307573)
2038403..2038969	PHAGE_Salmon_Fels_2_NC_010463: DNA-invertase; BN49_RS11400; phage(gi169936026)
2039112..2040284	PHAGE_Salmon_Fels_2_NC_010463: major tail sheath protein; BN49_RS11405; phage(gi169936025)
2040294..2040809	PHAGE_Salmon_Fels_2_NC_010463: major tail tube protein; BN49_RS11410; phage(gi169936024)
2040864..2041166	PHAGE_Salmon_Fels_2_NC_010463: P2 gpE-like tail protein; BN49_RS11415; phage(gi169936023)
2041181..2041300	PHAGE_Salmon_Fels_2_NC_010463: P2 gpE-like protein; BN49_RS11420; phage(gi169936022)
2041293..2044370	PHAGE_Salmon_Fels_2_NC_010463: P2 gpT-like tail protein; BN49_RS11425; phage(gi169936021)
2044367..2044852	PHAGE_Salmon_Fels_2_NC_010463: P2 gpU-like tail protein; BN49_RS11430; phage(gi169936020)
2044849..2045949	PHAGE_Salmon_Fels_2_NC_010463: tail protein; BN49_RS11435; phage(gi169936019)
2046040..2046258	PHAGE_Salmon_Fels_2_NC_010463: P2 gpOgr-like protein (acttivation of late gene expression); BN49_RS28925; phage(gi169936018)
2046330..2046348	attR

Prophage 3

CD position	BLAST-HIT
2333792..2333804	attL
complement(2333867..2334994)	PHAGE_Entero_HK022_NC_002166: integrase; BN49_RS12785; phage(gi9634144)
complement(2334975..2335220)	excisionase; BN49_RS12790
complement(2335273..2337042)	PHAGE_Entero_mEp237_NC_019704: exonuclease; BN49_RS27990; phage(gi435439296)
complement(2337054..2338327)	PROPHAGE_Escher_CFT073: transposase insC; BN49_RS12805; phage(gi26249447)
2338293..2338382	holin; BN49_RS30085
complement(2338358..2338747)	PHAGE_Entero_mEp237_NC_019704: exonuclease; BN49_RS12815; phage(gi435439296)
complement(2338889..2339233)	transcriptional regulator; BN49_RS12820
complement(2339276..2339470)	hypothetical protein; BN49_RS12825
complement(2340313..2340702)	PHAGE_Entero_mEp237_NC_019704: prophage repressor; BN49_RS12830; phage(gi435439304)
2340804..2341019	PHAGE_Entero_mEp237_NC_019704: prophage anti-repressor; BN49_RS29005; phage(gi435439305)
2341022..2341576	PHAGE_Entero_mEp237_NC_019704: CII protein; BN49_RS12840; phage(gi435439306)
2341628..2342611	PHAGE_Entero_Tyrion_NC_031077: hypothetical protein; BN49_RS12845; phage(gi100043)
2342604..2343068	PHAGE_Entero_mEp237_NC_019704: DNA replication protein P; BN49_RS12850; phage(gi435439308)
2343082..2343522	PHAGE_Escher_Pollock_NC_027381: transcriptional regulator; BN49_RS12855; phage(gi849254852)
2343901..2344482	hypothetical protein; BN49_RS12860
2344485..2345039	hypothetical protein; BN49_RS12865
complement(2345462..2346166)	hypothetical protein; BN49_RS12870
2346519..2346752	PHAGE_Entero_mEp237_NC_019704: hypothetical protein; BN49_RS12875; phage(gi435439313)
2346764..2347054	PHAGE_Entero_mEp237_NC_019704: hypothetical protein; BN49_RS29010; phage(gi435439314)
2347095..2347487	PHAGE_Shigel_SfIV_NC_022749: regulation protein; BN49_RS12880; phage(gi557307569)
2347688..2348719	PHAGE_Entero_mEp460_NC_019716: hypothetical protein; BN49_RS12885; phage(gi428782365)
2348732..2349079	PHAGE_Shigel_SfII_NC_021857: antiterminator protein Q; BN49_RS12890; phage(gi526244682)
complement(2349098..2349982)	hypothetical protein; BN49_RS12895
complement(2349992..2350504)	hypothetical protein; BN49_RS29015
2351457..2351693	PHAGE_Entero_cdtI_NC_009514: lysis protein; BN49_RS12900; phage(gi148609439)
2351671..2352201	PHAGE_Escher_TL_2011c_NC_019442: lysozyme; BN49_RS12905; phage(gi418487070)
2352234..2352710	PROPHAGE_Salmon_LT2: phage-tail assembly-like protein; BN49_RS12910; phage(gi16765210)

2352940..2353272	PHAGE_Klebsi_phiKO2_NC_005857: Gp60; BN49_RS29020; phage(gi46402146)
2353254..2353574	PHAGE_Klebsi_phiKO2_NC_005857: Gp61; BN49_RS12920; phage(gi46402147)
2353574..2353996	PHAGE_Klebsi_phiKO2_NC_005857: Gp62; BN49_RS12925; phage(gi46402148)
2354228..2354716	PHAGE_Entero_c_1_NC_019706: terminase small subunit; BN49_RS12930; phage(gi428781736)
2354716..2356818	PHAGE_Entero_c_1_NC_019706: terminase large subunit; BN49_RS12935; phage(gi428781737)
2356815..2357027	PHAGE_Entero_cdtI_NC_009514: hypothetical protein; BN49_RS12940; phage(gi148609385)
2357027..2358529	PHAGE_Entero_c_1_NC_019706: portal protein; BN49_RS12945; phage(gi428781739)
2358480..2360501	PHAGE_Entero_c_1_NC_019706: head maturation protease; BN49_RS12950; phage(gi428781740)
2360585..2360911	PHAGE_Entero_c_1_NC_019706: hypothetical protein; BN49_RS12955; phage(gi428781741)
2360904..2361179	PHAGE_Entero_c_1_NC_019706: hypothetical protein; BN49_RS12960; phage(gi428781742)
2361183..2361761	PHAGE_Entero_c_1_NC_019706: minor tail protein; BN49_RS12965; phage(gi428781743)
2361758..2362159	PHAGE_Entero_mEp237_NC_019704: minor tail protein U; BN49_RS12970; phage(gi435439276)
2362168..2362911	PHAGE_Entero_phi80_NC_021190: major tail protein; BN49_RS12975; phage(gi824479592)
2362922..2363350	PHAGE_Entero_mEp237_NC_019704: minor tail protein G; BN49_RS12980; phage(gi435439278)
2363371..2363685	PHAGE_Entero_mEp237_NC_019704: tail assembly protein GT; BN49_RS12985; phage(gi435439279)
2363669..2366812	PHAGE_Entero_phi80_NC_021190: tail length tape measure protein precursor; BN49_RS12990; phage(gi824479595)
2366817..2367164	PHAGE_Entero_mEp237_NC_019704: minor tail protein; BN49_RS12995; phage(gi435439281)
2367161..2367916	PHAGE_Entero_mEp237_NC_019704: minor tail protein L; BN49_RS13000; phage(gi435439282)
2367918..2368628	PHAGE_Entero_mEp390_NC_019721: minor tail protein; BN49_RS13005; phage(gi428782680)
2368660..2369250	PHAGE_Entero_mEp237_NC_019704: tail assembly protein I; BN49_RS13010; phage(gi435439284)
2369313..2378129	PHAGE_Klebsi_phiKO2_NC_005857: Gp21; BN49_RS13015; phage(gi46402107)
2378191..2379687	PHAGE_Klebsi_Sushi_NC_028774: tail fiber; BN49_RS13020; phage(gi971742192)
2390718..2390730	attR

Prophage 4

CD position	BLAST-HIT
complement(2713404..2713664)	PHAGE_Entero_mEp460_NC_019716: hypothetical protein; BN49_RS14705; phage(gi428782341)
complement(2713784..2713969)	hypothetical protein; BN49_RS14710
complement(2713966..2714628)	PHAGE_Salmon_SJ46_NC_031129: integrase/recombinase; BN49_RS14715; phage(gi100097)
complement(2714621..2714965)	hypothetical protein; BN49_RS14720
complement(2715093..2715878)	PHAGE_Burkho_KS9_NC_013055: chromosome partitioning protein gp35; BN49_RS14725; phage(gi255033718)
complement(2715878..2716177)	PHAGE_Entero_mEp390_NC_019721: hypothetical protein; BN49_RS14730; phage(gi428782699)
complement(2716510..2716716)	PHAGE_Entero_mEp390_NC_019721: hypothetical protein; BN49_RS29095; phage(gi428782700)
complement(2716945..2717592)	PHAGE_Entero_mEp390_NC_019721: prophage repressor; BN49_RS14740; phage(gi428782701)
2717697..2717894	PHAGE_Entero_mEp390_NC_019721: prophage anti-repressor; BN49_RS14745; phage(gi428782702)
2717920..2718381	PHAGE_Entero_mEp390_NC_019721: hypothetical protein; BN49_RS14750; phage(gi428782704)
2718443..2718625	PHAGE_Shigel_SfII_NC_021857: hypothetical protein; BN49_RS29955; phage(gi526244674)
2718619..2718798	PHAGE_Salmon_118970_sal3_NC_031940: terminase large subunit; BN49_RS29100; phage(gi100119)
2718788..2719726	PHAGE_Shigel_SfII_NC_021857: O protein family protein; BN49_RS14760; phage(gi526244675)
2719723..2720532	PHAGE_Salmon_SJ46_NC_031129: portal vertex protein; BN49_RS14765; phage(gi100122)
2720542..2720919	PHAGE_Salmon_118970_sal3_NC_031940: holin; BN49_RS14770; phage(gi100062)
2720932..2721912	PHAGE_Entero_mEp460_NC_019716: hypothetical protein; BN49_RS14775; phage(gi428782365)
2721926..2722504	hypothetical protein; BN49_RS14780

2723281..2723676	PHAGE_Entero_mEp390_NC_019721: putative holin; BN49_RS14785; phage(gi428782712)
2723663..2723944	PHAGE_Entero_mEp390_NC_019721: putative holin; BN49_RS14790; phage(gi428782713)
2723944..2724573	PHAGE_Salmon_118970_sal3_NC_031940: tail completion protein; BN49_RS14795; phage(gi100131)
2724581..2724856	PHAGE_Entero_mEp390_NC_019721: hypothetical protein; BN49_RS14800; phage(gi428782715)
2724807..2724989	PHAGE_Entero_mEp390_NC_019721: hypothetical protein; BN49_RS29105; phage(gi428782716)
2725139..2726014	hypothetical protein; BN49_RS14810
2726019..2726645	hypothetical protein; BN49_RS14815
2726780..2727214	hypothetical protein; BN49_RS14820
2727279..2727629	PHAGE_Entero_mEp235_NC_019708: putative HNH endonuclease; BN49_RS29110; phage(gi428781871)
2727787..2728284	PHAGE_Entero_mEp235_NC_019708: terminase small subunit; BN49_RS14830; phage(gi428781811)
2728288..2730039	PHAGE_Entero_mEp235_NC_019708: terminase large subunit; BN49_RS14835; phage(gi428781812)
2730187..2731413	PHAGE_Entero_SfV_NC_003444: portal protein; BN49_RS14845; phage(gi19548993)
2731406..2732005	PHAGE_Entero_SfV_NC_003444: capsid protease; BN49_RS14850; phage(gi19548994)
2732015..2733253	PHAGE_Entero_SfV_NC_003444: capsid; BN49_RS14855; phage(gi19548995)
2733331..2733648	PHAGE_Shigel_SfII_NC_021857: head-tail connector protein; BN49_RS14860; phage(gi526244642)
2733657..2733995	PHAGE_Entero_mEp234_NC_019715: head-tail connector II; BN49_RS14865; phage(gi428782261)
2733992..2734441	PHAGE_Entero_HK97_NC_002167: Gp10; BN49_RS14870; phage(gi9634169)
2734438..2734785	PHAGE_Entero_mEp390_NC_019721: hypothetical protein; BN49_RS14875; phage(gi428782673)
2734842..2735546	PHAGE_Entero_mEp234_NC_019715: major tail subunit; BN49_RS14880; phage(gi428782264)
2735577..2735981	PHAGE_Escher_HK75_NC_016160: tail assembly chaperone; BN49_RS14885; phage(gi356870688)
2735984..2736289	PHAGE_Entero_HK106_NC_019768: tail protein; BN49_RS14890; phage(gi428783291)
2736363..2736596	PHAGE_Entero_HK022_NC_002166: gp20; BN49_RS14895; phage(gi9634138)
2736657..2740046	PHAGE_Entero_BP_4795_NC_004813: putative tail component; BN49_RS14900; phage(gi157166052)
2740067..2740540	PHAGE_Pseudo_JBD44_NC_030929: putative head morphogenesis protein; BN49_RS14905; phage(gi100034)
2740527..2741003	PHAGE_Pseudo_PS_1_NC_029066: hypothetical protein; BN49_RS14910; phage(gi985757717)
2741016..2741396	PHAGE_Pseudo_YMC11/07/P54_PAE_BP_NC_030909: hypothetical protein; BN49_RS14915; phage(gi100020)
2741393..2744470	PHAGE_Pseudo_PS_1_NC_029066: tail protein; BN49_RS14920; phage(gi985757719)
2744543..2746696	PHAGE_Burkho_KS10_NC_011216: hypothetical protein; BN49_RS14925; phage(gi198449310)
2746709..2747443	PHAGE_Burkho_KS10_NC_011216: hypothetical protein; BN49_RS14930; phage(gi198449311)
complement(2747499..2748143)	hypothetical protein; BN49_RS14935
2748311..2748610	PHAGE_Cronob_phiES15_NC_018454: hypothetical protein; BN49_RS14940; phage(gi401817614)
2748562..2749150	PHAGE_Salmon_Fels_2_NC_010463: DNA-invertase; BN49_RS14945; phage(gi169936026)
2749476..2749712	PHAGE_Erwini_ENT90_NC_019932: DNA invertase-like protein; BN49_RS14950; phage(gi431810970)
complement(2749811..2750911)	PHAGE_Entero_mEp460_NC_019716: integrase; BN49_RS14955; phage(gi428782338)
